# Hedgehog Signaling in Mesothelioma: 2019 Status

**DOI:** 10.3389/fgene.2019.01121

**Published:** 2019-11-07

**Authors:** Emanuela Felley-Bosco

**Affiliations:** Laboratory of Molecular Oncology, Division of Thoracic Surgery, University Hospital Zurich, Zurich, Switzerland

**Keywords:** malignant pleural mesothelioma, hedgehog signaling, personalized medicine, hedgehog inhibition, Gli-1 inhibition, tumor-stroma cross talk, macrophage differentiation

Malignant pleural mesothelioma (MPM) is the most common type (about 80% reviewed in Leard and Broaddus, 2004) of malignant mesothelioma, a rapidly fatal and highly resilient tumor arising in the mesothelium, a tissue of mesodermal origins which covers many of the important internal organs (reviewed in (Yap et al., 2017). This cancer is mostly associated with exposure to asbestos (reviewed in Felley-Bosco and MacFarlane 2018).

Few years ago, we and others reported about hedgehog (Hh) signaling in a subset of MPM patients (Shi et al., 2012; Zhang et al., 2013; Meerang et al., 2016). We briefly discuss here whether, taking into account recent knowledge, it would be worth to consider these observations for mesothelioma therapy. We first consider data obtained using high throughput mesothelioma profiling studies (Bueno et al., 2016; Hmeljak et al., 2018), then we mention the caveats about successful hedgehog inhibition therapy in cancer (reviewed in Curran 2018) and finally we highlight novel aspects of hedgehog signaling in the context of immune signaling in cancer. Information about Hh signaling expression in cancers other than mesothelioma can be found in some recent reviews (Wu et al., 2017; Niyaz et al., 2019; Pietrobono et al., 2019;).

Canonical Hh core signaling include hedgehog ligands (sonic hedgehog, Shh; desert hedgehog, Dhh; indian hedgehog, Ihh) (reviewed in Petrova and Joyner 2014) which activate the G protein-coupled receptor Smoothened (Smo), upon binding to the transmembrane receptor Patched (Ptch) removing its inhibitory effect. Activation of Smo then leads to nuclear translocation of the Glioma associated protein (Gli) family of transcription factors and induction of Hh target genes such as *Gli1, Ptch*1 and Hedgehog interacting protein (*Hhip*). The latter competes with Ptch by binding to Hh ligands (Chuang and McMahon 1999), while cell-adhesion-molecule-related/downregulated by oncogenes (Cdon), bioregional Cdon-binding protein (Boc), and Growth Arrest Specific 1 (Gas1) positively regulate Hh signaling. Gli protein levels and activities are primarily regulated by Suppressor of Fused (Sufu) which is a negative regulator of mammalian Hh signaling (Cooper et al., 2005; Svard et al., 2006).

While the Hh signaling pathway is necessary for embryonic mesothelial development (Dixit et al., 2013) it is inactive in mesothelium (Shi et al., 2012). We were the first to show *SHH* gene expression in human MPM tumor tissues along with increased expression levels of *HHIP* and *GLI1* (Shi et al., 2012). High levels of *GLI-1* expression is significantly associated with worst overall survival in two independent cohorts of patients [(Shi et al., 2012) and analysis of mesothelioma TCGA (https://portal.gdc.cancer.gov/projects/TCGA-MESO) data using Progene2 (Goswami and Nakshatri 2014)]. In a recent high-throughput multi-omics analysis (Bueno et al., 2016), Hh signaling expression was enriched in sarcomatoid histotype, which is generally associated with worst overall survival. We also observed activation of Hh signaling during mesothelioma development in mice exposed to asbestos (Rehrauer et al., 2018). Interestingly, a recent pan-cancer analysis (de Reyniès et al., 2019) revealed that not only *GLI-1* (p = 0.019584614) but also *GLI-2* (p = 0.035063475) and *SHH* (p = 0.003447888) high expression have prognostic value in mesothelioma TCGA. Intriguingly, such correlation was not observed (de Reyniès et al., 2019) by analyzing two other datasets, where expression was based on array data instead of RNA-seq. The reasons for the discrepancy are not straightforward. However, in all datasets bad prognosis was associated with epithelial to mesenchymal transition and stemness.

Oncoprint analysis (www.cBioportal.org) of TCGA data (**Figure 1A**) shows that few mesothelioma patients bear *PTCH1* truncation mutations or *SUFU* deep deletions and there is a statistically significant co-occurrence between alterations of several components of Hh signaling (**Figure 1B**). Interestingly however, there is no strict correlation with ligands expression and the ligand with the highest differential level is *DHH*. Mutations in human and murine mesothelioma cell lines has also been reported (Lim et al., 2013) (Sneddon et al., 2017).

**Figure 1:**
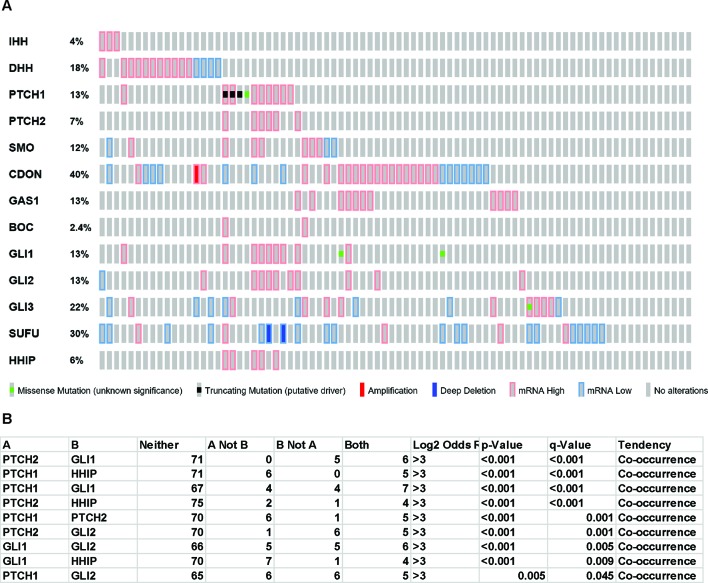
Hedgehog signaling in TCGA mesothelioma samples. “Oncoprint” analysis performed using cBioportal (www.cBioportal.org) of Hh components **(A)** and co-occurrence alterations statistically significant **(B)**.

As recently pointed out (Curran 2018), it has been not possible to maintain Hh signaling when primary tumors are grown in the presence of serum. However, we were able to culture primary human MPM, in 3% oxygen, in the absence of serum, but in the presence of their own conditioned medium plus specific growth factors and, in some of them, we showed an active Hh signaling (Shi et al., 2012). In these conditions we could observe (Felley-Bosco et al., 2015) the presence of primary cilia, a non-motile flagellar-like organelle present on growth-arrested cells (Satir et al., 2010) where Hh signaling occurs. This is possibly linked to the fact that about 35% of the cells grown in these conditions are quiescent (Renganathan et al., 2014), contrary to what is observed in cells cultured in the presence of serum. Giving the fact that in mesothelioma there is a considerable number of quiescent cells, since median cell-cycling marker Ki67 (also called MIB1) staining is 10% (Kadota et al., 2012), an additional advantage of these culture conditions is to better resemble to tumors. Interestingly, DHH is the ligand whose expression is maintained in these conditions in several human and rodent mesothelioma models (Shi et al., 2012) (Meerang et al., 2016). The reason for a differential expression of *DHH* in mesothelioma is not clear, especially because it is mostly associated with testis (Bitgood et al., 1996) and Schwann cell (Parmantier et al., 1999) development. An intriguing recent observation is that DHH positively regulates the differentiation from common myeloid progenitor (CMP) to granulocyte/macrophage progenitor and decreases the differentiation from CMP to megakaryocyte/erythrocyte progenitor (Lau et al., 2012). Are DHH producing mesothelioma cells influencing hematopoietic cells in the microenvironment?

Consistent with caveats recently discussed (Curran, 2018), it seems that Hh activation signature is not always associated with mutation in driver genes in mesothelioma although there are few patients with Hh driver mutations. Some Hh signature might be reflecting stromal activity. Pre-clinical studies have shown a moderate effect on tumor reduction accompanied by tumoral decrease of Hh-activation after treatment with Smo antagonists of tumor-bearing immunodeficient mice (Shi et al., 2012), while in another model in immunocompetent rats (Meerang et al., 2016) the tumor decreasing effect was associated with a marked effect on tumor stroma.

Patients with *PTCH1* driver mutations should respond to Smo inhibitors. The latter have been tested only in three unselected mesothelioma patients and no response was observed (LoRusso et al., 2011). As for other targeted therapy, there is a need for proper predictive biomarkers. In medulloblastoma, another cancer where a subgroup of patients shows Hh activation, a five-gene expression signature was used to select patients who received Smo inhibitor and 66% showed objective responses (Shou et al., 2015). In a more recent study, this Hh signature showed that the five responders and three patients with stable disease had Hh-activated tumors, while two patients with activated Hh and 50 patient with an Hh-negative signature did not respond to Smo inhibition (Kieran et al., 2017). It is still not known whether this gene signature would be the same in mesothelioma, and it is not sure whether energy will be invested in tackling Hh signaling as therapeutic strategy in few patients, in view of the more frequent signaling pathways altered in this disease (Bueno et al., 2016; Hmeljak et al., 2018).

Patients with Hh-ligand independent mutations may benefit from alternative therapeutic strategies. Several studies reported that Gli1 inhibition, either by agents such as arsenic trioxide, which prevents Gli2 localization to primary cilia (Kim et al., 2010) or GANT61, which prevents Gli1-DNA binding in living cells (Lauth et al., 2007), resulted in growth arrest and induction of cell death in MPM cell *in vitro* (Li et al., 2013; You et al., 2014; Lim et al., 2015).

Further complexity is added by the recent observation that Shh produced by tumor associated astrocytes promote medullobastoma growth by increasing nestin expression independently of Gli-1 (Liu et al., 2017). Nestin is a biomarker of epithelial to mesenchymal transition in MPM and high expression levels are associated with worst outcome (Thies et al., 2015), but it is not clear whether it is associated with any Hh signaling in MPM.

Finally, there is an aspect of Hh signaling which is worth mentioning in the context of immunotherapy, which is changing the handling of cancer patients, including MPM patients. Hh ligands (including DHH) produced by tumor cells lead to Gli-1 mediated “M2-polarization” of macrophages (Hanna et al., 2019) which is associated with immunosuppression and pro-tumorigenic activity. This additional cross talk between tumor cells and stroma is therefore of potential importance in a cancer characterized by abundant “M2-polarized” macrophages (Minnema-Luiting et al., 2018).

Dr. Curran mentioned (Curran 2018) that the difference between Hh inhibitor concentration leading to the decrease of hedgehog reporter activity in mouse fibroblast and the concentration necessary to inhibit tumor cell growth is indicating reasons for clinical failure in the treatment of cancer patients using Hh inhibition.

Although agreeing with that caveat, based on our own and recent data in multi-omics studies, our opinion is that it is likely that Hh signaling functions as a pro-tumorigenic signal in some MPM. Indeed, both Hh ligand-dependent and ligand-independent effects promote MPM cell growth in experimental models and high through-put analysis of MPM patients shows that the Hh pathway is active in a subset of patients. It is up to the mesothelioma research community to coordinate efforts to further investigate this aspect.

## Author Contributions

The author confirms being the sole contributor of this work and approved it for publication.

## Funding

This work was supported by the Swiss National Science Foundation grant 320030_182690 and the Stiftung für Angewandte Krebsforchung.

## Conflict of Interest

The author declares that the research was conducted in the absence of any commercial or financial relationships that could be construed as a potential conflict of interest.

## References

[B1] Bitgood M. J. Shen L. McMahon A. P. (1996). Sertoli cell signaling by Desert hedgehog regulates the male germline. Curr. Biol. 6 (3), 298–304. 10.1016/S0960-9822(02)00480-3 8805249

[B2] Bueno R. Stawiski E. W. Goldstein L. D. Durinck S. De Rienzo A. Modrusan Z. (2016). Comprehensive genomic analysis of malignant pleural mesothelioma identifies recurrent mutations, gene fusions and splicing alterations. Nat. Genet. 48 (4), 407–416. 10.1038/ng.3520 26928227

[B3] Chuang P. T. McMahon A. P. (1999). Vertebrate Hedgehog signalling modulated by induction of a Hedgehog-binding protein. Nature 397 (6720), 617–621. 10.1038/17611 10050855

[B4] Cooper A. F. Yu K. P. Brueckner M. Brailey L. L. Johnson L. McGrath J. M. (2005). Cardiac and CNS defects in a mouse with targeted disruption of suppressor of fused. Development 132 (19), 4407–4417. 10.1242/dev.02021 16155214

[B5] Curran T. (2018). Reproducibility of academic preclinical translational research: lessons from the development of Hedgehog pathway inhibitors to treat cancer. Open Biol. 8 (8), 180098. 10.1098/rsob.180098 30068568 PMC6119869

[B6] de Reyniès A. Javelaud D. Elarouci N. Marsaud V. Gilbert C. Mauviel A. (2019). Large-scale pan-cancer analysis reveals broad prognostic association between TGF-β ligands, not Hedgehog, and GLI1/2 expression in tumors. BioRxiv. 10.1101/728949 PMC7468122 32879407

[B7] Dixit R. Ai X. Fine A. (2013). Derivation of lung mesenchymal lineages from the fetal mesothelium requires hedgehog signaling for mesothelial cell entry. Development 140 (21), 4398–4406. 10.1242/dev.098079 24130328 PMC4007715

[B8] Felley-Bosco E. MacFarlane M. (2018). Asbestos: modern insights for toxicology in the era of engineered nanomaterials. Chem. Res. Toxicol. 31 994–1008. 10.1021/acs.chemrestox.8b00146 30156102

[B9] Felley-Bosco E. Opitz I. Meerang M. (2015). Hedgehog signaling in malignant pleural mesothelioma. Genes (Basel) 6 (3), 500–511. 10.3390/genes6030500 26184317 PMC4584313

[B10] Goswami C. P. Nakshatri H. (2014). PROGgeneV2: enhancements on the existing database. BMC Cancer 14, 970. 10.1186/1471-2407-14-970 25518851 PMC4300843

[B11] Hanna A. Metge B. J. Bailey S. K. Chen D. Chandrashekar D. S. Varambally S. (2019). Inhibition of Hedgehog signaling reprograms the dysfunctional immune microenvironment in breast cancer. Oncoimmunology 8 (3), 1548241. 10.1080/2162402X.2018.1548241 30723576 PMC6350695

[B12] Hmeljak J. Sanchez-Vega F. Hoadley K. A. Shih J. Stewart C. Heiman D. (2018). Integrative molecular characterization of malignant pleural mesothelioma. Cancer Discovery 8 (12), 1548–1565. 10.1158/2159-8290.CD-18-0804 30322867 PMC6310008

[B13] Kadota K. Suzuki K. Colovos C. Sima C. S. Rusch V. W. Travis W. D. (2012). A nuclear grading system is a strong predictor of survival in epitheloid diffuse malignant pleural mesothelioma. Mod. Pathol. 25 (2), 260–271. 10.1038/modpathol.2011.146 21983936 PMC4080411

[B14] Kieran M. W. Chisholm J. Casanova M. Brandes A. A. Aerts I. Bouffet E. (2017). Phase I study of oral sonidegib (LDE225) in pediatric brain and solid tumors and a phase II study in children and adults with relapsed medulloblastoma. Neuro Oncol. 19 (11), 1542–1552. 10.1093/neuonc/nox109 28605510 PMC5737275

[B15] Kim J. Lee J. J. Kim J. Gardner D. Beachy P. A. (2010). Arsenic antagonizes the Hedgehog pathway by preventing ciliary accumulation and reducing stability of the Gli2 transcriptional effector. Proc. Natl. Acad. Sci. U.S.A. 107 (30), 13432–13437. 10.1073/pnas.1006822107 20624968 PMC2922148

[B16] Lau C. I. Outram S. V. Saldana J. I. Furmanski A. L. Dessens J. T. Crompton T. (2012). Regulation of murine normal and stress-induced erythropoiesis by Desert Hedgehog. Blood 119 (20), 4741–4751. 10.1182/blood-2011-10-387266 22461491 PMC3375146

[B17] Lauth M. Bergstrom A. Shimokawa T. Toftgard R. (2007). Inhibition of GLI-mediated transcription and tumor cell growth by small-molecule antagonists. Proc. Natl. Acad. Sci. U.S.A. 104 (20), 8455–8460. 10.1073/pnas.0609699104 17494766 PMC1866313

[B18] Leard L. E. Broaddus V. C. (2004). Mesothelial cell proliferation and apoptosis. Respirology 9 (3), 292–299. 10.1111/j.1440-1843.2004.00602.x 15362999

[B19] Li H. Lui N. Cheng T. Tseng H. H. Yue D. Giroux-Leprieur E. (2013). Gli as a novel therapeutic target in malignant pleural mesothelioma. PloS One 8 (3), e57346. 10.1371/journal.pone.0057346 23483902 PMC3590216

[B20] Lim C. B. Prele C. M. Baltic S. Arthur P. G. Creaney J. Watkins D. N. (2015). Mitochondria-derived reactive oxygen species drive GANT61-induced mesothelioma cell apoptosis. Oncotarget 6 (3), 1519–1530. 10.18632/oncotarget.2729 25544756 PMC4359311

[B21] Lim C. B. Prele C. M. Cheah H. M. Cheng Y. Y. Klebe S. Reid G. (2013). Mutational analysis of hedgehog signaling pathway genes in human malignant mesothelioma. PloS One 8 (6), e66685. 10.1371/journal.pone.0066685 23826113 PMC3691204

[B22] Liu Y. Yuelling L. W. Wang Y. Du F. Gordon R. E. O'Brien J. A. (2017). Astrocytes promote medulloblastoma progression through hedgehog secretion. Cancer Res. 77 (23), 6692–6703. 10.1158/0008-5472.CAN-17-1463 28986380 PMC5759326

[B23] LoRusso P. M. Rudin C. M. Reddy J. C. Tibes R. Weiss G. J. Borad M. J. (2011). Phase I trial of hedgehog pathway inhibitor vismodegib (GDC-0449) in patients with refractory, locally advanced or metastatic solid tumors. Clin. Cancer Res. 17 (8), 2502–2511. 10.1158/1078-0432.CCR-10-2745 21300762 PMC5244484

[B24] Meerang M. Berard K. Felley-Bosco E. Lauk O. Vrugt B. Boss A. (2016). Antagonizing the hedgehog pathway with vismodegib impairs malignant pleural mesothelioma growth in vivo by affecting stroma. Mol. Cancer Ther. 15 (5), 1095–1105. 10.1158/1535-7163.MCT-15-0583 26839306

[B25] Minnema-Luiting J. Vroman H. Aerts J. Cornelissen R. (2018). Heterogeneity in immune cell content in malignant pleural mesothelioma. Int. J. Mol. Sci. 19, 38–49. 10.3390/ijms19041041 PMC5979422 29601534

[B26] Niyaz M. Khan M. S. Mudassar S. (2019). Hedgehog signaling: an achilles' heel in cancer. Transl. Oncol. 12 (10), 1334–1344. 10.1016/j.tranon.2019.07.004 31352196 PMC6664200

[B27] Parmantier E. Lynn B. Lawson D. Turmaine M. Namini S. S. Chakrabarti L. (1999). Schwann cell-derived Desert hedgehog controls the development of peripheral nerve sheaths. Neuron 23 (4), 713–724. 10.1016/S0896-6273(01)80030-1 10482238

[B28] Petrova R. Joyner A. L. (2014). Roles for Hedgehog signaling in adult organ homeostasis and repair. Development 141 (18), 3445–3457. 10.1242/dev.083691 25183867 PMC4197719

[B29] Pietrobono S. Gagliardi S. Stecca B. (2019). Non-canonical hedgehog signaling pathway in cancer: activation of gli transcription factors beyond smoothened. Front. Genet. 10, 556. 10.3389/fgene.2019.00556 31244888 PMC6581679

[B30] Rehrauer H. Wu L. Blum W. Pecze L. Henzi T. Serre-Beinier V. (2018). How asbestos drives the tissue towards tumors: YAP activation, macrophage and mesothelial precursor recruitment, RNA editing, and somatic mutations. Oncogene 37, 2645–2659. 10.1038/s41388-018-0153-z 29507420 PMC5955862

[B31] Renganathan A. Kresoja-Rakic J. Echeverry N. Ziltener G. Vrugt B. Opitz I. (2014). GAS5 long non-coding RNA in malignant pleural mesothelioma. Mol. Cancer 13 (1), 119. 10.1186/1476-4598-13-119 24885398 PMC4039656

[B32] Satir P. Pedersen L. B. Christensen S. T. (2010). The primary cilium at a glance. J. Cell Sci. 123 (Pt 4), 499–503. 10.1242/jcs.050377 20144997 PMC2818190

[B33] Shi Y. Moura U. Opitz I. Soltermann A. Rehrauer H. Thies S. (2012). Role of hedgehog signaling in malignant pleural mesothelioma. Clin. Cancer Res. 18 (17), 4646–4656. 10.1158/1078-0432.CCR-12-0599 22733539

[B34] Shou Y. Robinson D. M. Amakye D. D. Rose K. L. Cho Y. J. Ligon K. L. (2015). A five-gene hedgehog signature developed as a patient preselection tool for hedgehog inhibitor therapy in medulloblastoma. Clin. Cancer Res. 21 (3), 585–593. 10.1158/1078-0432.CCR-13-1711 25473003

[B35] Sneddon S. Patch A. M. Dick I. M. Kazakoff S. Pearson J. V. Waddell N. (2017). Whole exome sequencing of an asbestos-induced wild-type murine model of malignant mesothelioma. BMC Cancer 17 (1), 396. 10.1186/s12885-017-3382-6 28577549 PMC5455120

[B36] Svard J. Heby-Henricson K. Persson-Lek M. Rozell B. Lauth M. Bergstrom A. (2006). Genetic elimination of Suppressor of fused reveals an essential repressor function in the mammalian Hedgehog signaling pathway. Dev. Cell 10 (2), 187–197. 10.1016/j.devcel.2005.12.013 16459298

[B37] Thies S. Friess M. Frischknecht L. Korol D. Felley-Bosco E. Stahel R. (2015). Expression of the stem cell factor nestin in malignant pleural mesothelioma is associated with poor prognosis. PloS One 10 (9), e0139312. 10.1371/journal.pone.0139312 26421614 PMC4589394

[B38] Wu F. Zhang Y. Sun B. McMahon A. P. Wang Y. (2017). Hedgehog signaling: from basic biology to cancer therapy. Cell Chem. Biol. 24 (3), 252–280. 10.1016/j.chembiol.2017.02.010 28286127 PMC7442121

[B39] Yap T. A. Aerts J. G. Popat S. Fennell D. A. (2017). Novel insights into mesothelioma biology and implications for therapy. Nat. Rev. Cancer 17 (8), 475–488. 10.1038/nrc.2017.42 28740119

[B40] You M. Varona-Santos J. Singh S. Robbins D. J. Savaraj N. Nguyen D. M. (2014). Targeting of the Hedgehog signal transduction pathway suppresses survival of malignant pleural mesothelioma cells in vitro. J. Thorac. Cardiovasc. Surg. 147 (1), 508–516. 10.1016/j.jtcvs.2013.08.035 24094913

[B41] Zhang Y. He J. Zhang F. Li H. Yue D. Wang C. (2013). SMO expression level correlates with overall survival in patients with malignant pleural mesothelioma. J. Exp. Clin. Cancer Res. 32, 7. 10.1186/1756-9966-32-7 23379358 PMC3622612

